# Higher expression of TSR2 aggravating hypertension via the PPAR signaling pathway

**DOI:** 10.18632/aging.205852

**Published:** 2024-05-29

**Authors:** Ling-Bing Meng, Gai-Feng Hu, Tingting Lv, Changhua Lv, Lianfeng Liu, Ping Zhang

**Affiliations:** 1 Department of Cardiology, Beijing Tsinghua Changgung Hospital, School of Clinical Medicine, Tsinghua University, Beijing, China; 2 Department of Cardiology, Beijing Anzhen Hospital, Capital Medical University, National Clinical Research Center for Cardiovascular Diseases, Chaoyang 100029, Beijing, China

**Keywords:** hypertension, TSR2, PPAR, hub genes, biomarker

## Abstract

Hypertension is a complex disease with unknown causes. Therefore, it’s crucial to deeply study its molecular mechanism. The hypertension dataset was obtained from Gene Expression Omnibus data base (GEO), and miRNA regulating central hub genes was screened via weighted gene co-expression network (DEGs) and gene set enrichment (GSEA). Cell experiments validated TSR2’s role and the PPAR signaling pathway through western blotting. 500 DEGs were identified for hypertension, mainly enriched in actin cross-linking, insulin signaling, PPAR signaling, and protein localization. Eight hub genes (SEC61G, SRP14, Liy AR, NIP7, SDAD1, POLR1D, DYNLL2, TSR2) were identified. Four hub genes (LYAR, SDAD1, POLR1D, TSR2) exhibited high expression levels in the hypertensive tissue samples, while showing low expression levels in the normal tissue samples. This led us to speculate that they may have relevant regulatory effects on hypertension. When TSR2 was knocked down in the hypertension peripheral blood mononuclear cells (PBMC) model, the critical proteins in the PPAR signaling pathway (FABP, PPAR, PLTP, ME1, SCD1, CYP27, FABP1, OLR1, CPT-1, PGAR, CAP, ADIPO, MMP1, UCP1, ILK, PDK1 UBC AQP7) were downregulated. This also occurred in the hypertension peripheral blood mononuclear cells (PBMC) + TSR2_ OV model. TSR2 is highly expressed in individuals with hypertension and may play a significant role in the development of hypertension through the PPAR signaling pathway. TSR2 could serve as a molecular target for the early diagnosis and precise treatment of hypertension, providing a valuable direction for the mechanism research of this condition.

## INTRODUCTION

Hypertension is a highly complex disease with its pathophysiological characteristics interfered and impacted by various of factors, resulting in widespread and persistent hypertension and related complications [1]. There are significant differences in the clinical characteristics of hypertension among different age groups, and attention should be paid to the features of hypertension during menopause, such as a relatively increased pulse pressure, a large fluctuation range of blood pressure, and abnormal glucose and lipid metabolism [2]. In about 95 percent of hypertension cases, the cause is unknown, and the exact mechanism of this type of hypertension remains unclear. Therefore, it is important to conduct a thorough study of the molecular mechanism of hypertension [3].

TSP-1 contains three primer-like motifs (TSR1, TSR2, TSR3). The KRFK amino acid domain between TSR1 and TSR2 specifically binds to L-TGF-β1, while the CSVTCG amino acid domain in TSR2 and TSR3 specifically binds to the TSP-1 receptor CD36. Only these three motifs exhibit specific binding to the TSP-1 receptor. Under the action of biochemical enzymes *in vivo*, activated TGF-β1 can be produced, subsequently playing a role in vascular fibrosis [4–8].

TSR2 (Threonyl-tRNA Synthetase 2, Mitochondrial) is a gene encoding a specific protein typically located in the mitochondria of cells. Mitochondria, crucial organelles within cells, play a primary role in generating cellular energy through the oxidation of carbohydrates and fats, resulting in the production of adenosine triphosphate (ATP). TSR2 is frequently associated with processes such as microvascular endothelial migration, microvascular endothelial cell tube formation, and vascular budding, particularly within the aortic arch. Notably, mutations in TSR2, as opposed to TSR3, significantly diminish anti-angiogenic activity within the Arg-Trp ladder of TSR2 [9]. Thrombospondin 1 (TSP1) features Type 1 (TSR1) and Type 2 (TSR2) repeat sequences that share partial sequence homology. Both TSP1 and von Willebrand factor (VWF) engage with the platelet GPIb/V/IX membrane complex, especially under flow conditions. These interactions regulate platelet recruitment to (sub)endothelial VWF and TSP1, which become exposed in the circulatory system due to vascular inflammation and endothelial injury. Similarly, TSR2 serves a comparable function, demonstrating pro-inflammatory and pro-thrombotic properties [10].

High blood pressure is characterized by elevated pressure exerted by the blood on the arterial walls during circulation. In severe cases, it can lead to cardiovascular and cerebrovascular diseases, including the formation of blood clots. Peroxisome proliferator-activated receptor (PPAR) is a ligand-activated nuclear transcription factor belonging to the nuclear receptor superfamily. Upon binding to its ligand, PPAR is activated, inducing the expression of a multitude of target genes [11]. Rubattu [12] demonstrated that the early expression of the antioxidant AMPK/PPARα/UCP2 pathway significantly impacts hypertensive mice, especially in the context of severe hypertension, where spontaneously hypertensive stroke-prone (SHRSP) rats are more susceptible to oxidative stress-dependent target organ damage.

Currently, the majority of research predominantly centers on unraveling the molecular mechanisms that link TSP1 to cardiovascular and cerebrovascular diseases. In contrast, the association between TSR2 and hypertension remains elusive. Furthermore, there is a significant scarcity of literature reporting the specific mechanistic role of TSR2 through the PPAR pathway in the development and progression of hypertension.

With the rapid development of high-throughput sequencing technology, gene expression analysis using gene chips has emerged as a reliable method for identifying genomic changes in the occurrence and development of diseases [13]. The high-throughput gene expression database contains a wealth of rich disease gene chip data, offering an extensive repository of gene expression profiles. This serves as a foundational resource for the investigation of genomic changes related to hypertension and the exploration of its core differential expressions [14, 15].

Therefore, this study screened out hypertension-related chip data from the Gene Expression Omnibus data base (GEO) database, utilized bioinformatics technology to identify core genes distinguishing hypertensive individuals from normal individuals, and performed enrichment analysis and pathway analysis. The significant role of TSR2 and PPAR signaling pathway in hypertension was validated using public datasets, further confirmed by basic experiment. The aim is to explore specific biomarkers and potential therapeutic targets for hypertension, and providing a theoretical basis for a deeper understanding of the pathogenesis of hypertension.

## MATERIALS AND METHODS

### Hypertension data set

In this study, high blood pressure dataset GSE75672 configuration file generated from the GPL10558 Gene Expression Omnibus data base (GEO) database (http://www.ncbi.nlm.nih.gov/geo/) was download. GSE75672 comprises 10 hypertension and 11 normal tissue samples to identify differentially expressed genes (DEGs) associated with hypertension.

### Screening of differentially expressed genes (DEGs)

We used the R software package limma (version3.40.6) for difference analysis. The expression spectrum data set of GSE75672 was obtained. The lmFit function was employed for multiple linear regression, followed by the use of the eBays function. Through empirical Bayesian adjustment of the standard error toward a common value, the regulated t statistic, the regulated f statistic and the logarithmic ratio of the differential expression were calculated, and the difference significance of each gene was finally obtained, and the volcano map was made to obtain differentially expressed genes (DEGs).

### Weighted gene coexpression network analysis (WGCNA)

Firstly, we calculated the Median Absolute Deviation (MAD) for each gene using the GSE75672 expression profile. The top 50% of genes with the minimum Median Absolute Deviation (MAD) values were then excluded. Outliers and samples were removed using good Samples Genes method of R software package Weighted Gene Coexpression Network Analysis (WGCNA). Subsequently, a scale-free co-expression network was constructed using Weighted Gene Coexpression Network Analysis (WGCNA). Initially, the Pearson correlation matrix and mean linkage method were applied to all pairs of genes. Then, the power function A_mn = | C_mn | ^ beta structure was employed to create a weighted adjacency matrix (where C_mn represents the Pearson correlation between Gene_n; A_mn represents the adjacency between Genem and Genen). β is a soft threshold parameter, which can emphasize the strong correlation between genes and weaken the influence of weak correlation and negative correlation. After selecting a power of 10, the adjacency matrix was transformed into a topological overlap matrix (TOM). This matrix measures the network connectivity of a gene, defined as the sum of its adjacency to all other genes, and is used for network gene ratios. The corresponding dissimilarity (1-TOM) was calculated. To classify genes with similar expression profiles into modules, we employed average linkage hierarchical clustering based on the topological overlap matrix (TOM) based dissimilarity measure, setting the minimum size (genome) of the gene tree map to 30 and sensitivity to 3. To further analyze the modules, we calculated the heterogeneity of the characteristic genes of the modules, selected a cut line for the module tree, and merged some modules. It is worth noting that grey modules are considered collections of genes that cannot be assigned to any module.

### Construction and analysis of protein-protein interaction (PPI) network

The STRING database is designed to collect, score, and integrate all publicly available sources of information on protein-protein interactions, and supplement these sources with computational predictions. In this study, the list of differentially expressed genes was input into the Search Tool for the Retrieval of Interacting Genes (STRING) database to construct a protein-protein interaction (PPI) network for predicting core genes (confidence >0.4). Cytoscape software enables biological network analysis and two-dimensional (2D) visualization for biologists. In this study, Cytoscape software was used to visualize and predict core genes in protein-protein interaction (PPI) network formed by string database. First, the protein-protein interaction (PPI) network was imported into Cytoscape software, and the modules with the best correlation were identified through MCODE. Additionally, 10 genes with the highest correlation were calculated using two algorithms (MCC and MNC), and the list of core genes is derived after visualization.

### Functional enrichment analysis

Gene Ontology Analysis (GO) and the Kyoto Encyclopedia of Genes and Genomes (KEGG) analysis are computational methods used to assess gene function and biological pathways. In this study, we utilized the Kyoto Encyclopedia of Genes and Genomes analysis (KEGG) rest API (https://www.kegg.jp/kegg/rest/keggapi.html) to obtain the latest Kyoto Encyclopedia of Genes and Genomes analysis (KEGG) Pathway gene annotation by inputting the gene list. Subsequently, genes were mapped into the background set, and enrichment analysis was conducted using the R software package clusterProfiler (version 3.14.3) to obtain the enrichment results of the gene set. The Gene Ontology Analysis (GO) annotation of genes in R software package org. Hs.eg.db (version 3.1.0) was also used as the background to map the genes into the background set. The minimum gene set was set as 5 and the maximum gene set as 5000. *P*-value of < 0.05 and a false discovery rate (FDR) of <0.25 were considered statistically significant measures.

In addition, the Metascape database provides a comprehensive resource for annotating and analyzing gene lists, with visual export. We use the Metascape database (http://metascape.org/gp/index.html), for the above differences in gene enrichment of function analysis and export list.

### Gene set enrichment

For Gene set enrichment analysis (GSEA, http://software.broadinstitute.org/gsea/index.jsp), we divided the sample into two groups based on hypertension and normal tissue, and derived Molecular Signatures. Database (https://doi.org/10.1093/bioinformatics/btr260) to download C2. Cp. Kegg. V7.4. Symbols. GMT collections. This allowed us to review the related pathways and molecular mechanisms based on gene expression and phenotype grouping. We set the minimum gene sets at 5 and the maximum gene sets at 5000, with one thousand heavy sampling. Statistical significance was determined by a *P*-value of < 0.05 and a false discovery rate (FDR) of <0.25.

### Gene expression heat map

We used R-packet heatmap to make a heatmap of the expression levels of core genes found by the two algorithms in the protein-protein interaction (PPI) network in GSE75672, and visualized the expression differences of core genes between hypertension and normal tissue samples.

### Comparative toxicogenomics database analysis

The Comparative Toxicogenomics Database integrates a large number of chemicals, genetic, and functional data, providing significant convenience for research into the potential mechanisms of disease and drug-related exposures. We put the core genes into the Comparative Toxicogenomics Database (CTD) website to identify diseases most related to the core genes. Additionally, we used Excel to create a radar map illustrating the expression differences of each gene.

### miRNA

TargetScan (https://www.targetscan.org/) is an online database for predictive analysis of miRNA and target genes. In our study, TargetScan was employed to screen for miRNA that regulates central DEG.

### Cell culture

In this study, peripheral blood mononuclear cells (PBMC) were collected from 4–6-week-old spontaneously hypertensive rats (SHR), which serve as a model of spontaneous hypertension. The control group consisted of peripheral blood mononuclear cells (PBMC) obtained from the peripheral blood of wild-type 4–6-week-old SD rats.

#### Isolation of peripheral blood mononuclear cells (PBMC)

Collect the peripheral blood sample using appropriate anticoagulant (e.g., heparin or EDTA). Dilute the blood sample with a balanced salt solution (e.g., phosphate-buffered saline, PBS) at a 1:1 ratio. Layer the diluted blood sample onto a density gradient medium such as Ficoll-Paque in a centrifuge tube. Centrifuge the tube at a low speed to separate the peripheral blood mononuclear cells (PBMC) from other blood components (e.g., red blood cells). Carefully collect the peripheral blood mononuclear cells (PBMC) layer from the gradient interface and transfer it to a new centrifuge tube. Wash the collected peripheral blood mononuclear cells (PBMC) by adding PBS and centrifuging them to remove any remaining density gradient medium.

#### Cell culture

Count the isolated peripheral blood mononuclear cells (PBMC) using a hemocytometer or an automated cell counter.

Plate the cells at the desired cell density in tissue culture flasks or plates. A common starting cell density is around 1-2 million cells per milliliter. Use a suitable cell culture medium, such as RPMI-1640, supplemented with 10% FBS and antibiotics (penicillin-streptomycin). Optionally, add specific growth factors, cytokines, or other supplements based on your experimental needs. Culture the cells in a humidified CO_2_ incubator at 37°C. Maintain the appropriate CO_2_ level (usually 5%) to maintain physiological pH.

Regularly check the cells under a microscope for attachment, morphology, and overall health.

#### Subculturing (Passaging)

As the peripheral blood mononuclear cells (PBMC) proliferate, they will reach confluence in the culture vessel. To prevent overgrowth and maintain optimal conditions, it is necessary to subculture the cells by splitting them into new culture vessels. Detach the cells using trypsin or other cell detachment reagents according to your laboratory’s protocol, count the cells, and plate them at the desired cell density in new culture vessels with fresh medium.

#### Harvesting cells for experiments

Once the peripheral blood mononuclear cells (PBMC) have reached the desired stage of growth, you can harvest them for experiments. Detach the cells as mentioned earlier and collect them in a tube. Centrifuge the cells to form a pellet.

Remove the supernatant and proceed with downstream experiments or analyses.

Remember that the exact protocols may vary based on the specific requirements of your experiments and the guidelines of your laboratory. Always maintain sterile techniques, adhere to ethical considerations, and work in a biosafety cabinet when necessary.

### Cell groups and genetic intervention

The peripheral blood mononuclear cells were divided into four groups: control peripheral blood mononuclear cells (PBMC), hypertension peripheral blood mononuclear cells (PBMC), hypertension peripheral blood mononuclear cells (PBMC) + TSR2_knock down (KD), hypertension peripheral blood mononuclear cells (PBMC) + TSR2_over expression (OV).

### The procedure of TSR2_knock down in the hypertension peripheral blood mononuclear cells

#### Designing short hairpin RNA (shRNA)

Identify the target sequence within the TSR2 gene that you want to silence. This sequence should be specific to TSR2 and not have significant homology with other genes. Design a short hairpin RNA (shRNA) sequence that corresponds to the target TSR2 sequence. The shRNA will trigger the RNA interference (RNAi) machinery to degrade the TSR2 mRNA. Consider using software tools that specialize in RNAi target prediction to ensure the effectiveness and specificity of your shRNA design.

#### Constructing RNAi vectors

Clone the designed shRNA sequence into a suitable RNAi vector. Commonly employed vectors include those based on the Agrobacterium tumefaciens binary system or viral vectors. Ensure that your vector includes appropriate promoters to drive shRNA expression.

#### Transformation

Introduce the constructed RNAi vector into hypertension peripheral blood mononuclear cells (PBMC) using suitable transformation methods, such as Agrobacterium-mediated transformation or biolistic particle delivery (gene gun). Select transformed plant cells using appropriate selectable markers present in the vector.

#### Confirmation of knockdown

Analyze the transgenic plants to confirm the knockdown of TSR2 expression. Western blot analysis or immunodetection assays can be employed to assess the reduction in TSR2 protein levels.

### The procedure of TSR2_over expression in the hypertension peripheral blood mononuclear cells

#### Vector construction

Design an appropriate expression vector for overexpressing TSR2. This vector should include strong promoters to drive gene expression, such as a constitutive promoter or a promoter specific to the tissue of interest. Clone the TSR2 coding sequence into the expression vector, ensuring that the TSR2 coding sequence is properly oriented and in-frame with the chosen promoter.

#### Plasmid amplification

Amplify the constructed plasmid in bacteria using standard molecular biology techniques and purify the plasmid DNA using methods such as mini-prep or maxi-prep kits.

Select cells that have successfully taken up and expressed the TSR2 overexpression construct, typically achieved by incorporating a selectable marker into the vector.

#### Stable cell line generation

To guarantee consistent and enduring overexpression of TSR2, establish stable cell lines by meticulously selecting and cultivating cells exhibiting the highest levels of expression throughout multiple passages.

#### Confirmation of overexpression

Perform Western blot analysis to assess the elevation of TSR2 protein levels.

### The detection of PPAR signaling pathway

After interfering with the expression of TSR2 in the hypertension peripheral blood mononuclear cells (PBMC), the western blotting was used to detect the PPAR signaling pathway.

Sample Preparation: Begin by isolating and extracting proteins from your samples using appropriate lysis buffers and techniques. Next, denature the proteins by boiling them with a reducing agent and SDS loading buffer. Electrophoresis (SDS-PAGE): Load the denatured protein samples onto an SDS-PAGE gel, including molecular weight markers. Run electrophoresis to effectively separate proteins based on their molecular weight. Protein Transfer: Assemble a transfer stack with a gel, a membrane (nitrocellulose or PVDF), and filter papers soaked in transfer buffer. Transfer the separated proteins from the gel to the membrane using a suitable transfer apparatus. Apply an electric current to facilitate the transfer of proteins to the membrane. Membrane Blocking: Block the membrane with a blocking solution to prevent non-specific binding of antibodies. Incubate the membrane in the blocking solution for approximately 1 hour. Primary Antibody Incubation: Incubate the membrane with a primary antibody specific to the target protein (TSR2, FABP, PPAR, PLTP, ME1, SCD1, CYP27, FABP1, OLR1, CPT-1, PGAR, CAP, ADIPO, MMP1, UCP1, ILK, PDK1 UBC AQP7). Dilute the primary antibody in a suitable buffer containing blocking solution. Incubate overnight or for a few hours at room temperature or 4°C with gentle agitation. Washing: Perform several washes of the membrane with a washing buffer to remove unbound primary antibody. Secondary Antibody Incubation: Incubate the membrane with a secondary antibody conjugated to an enzyme (e.g., HRP). The secondary antibody recognizes the primary antibody and adds an enzymatic tag. Incubate for about 1 hour at room temperature with gentle agitation. Washing: Wash the membrane again to remove any unbound secondary antibody. Signal Detection: Initiate an enzymatic reaction by applying a chemiluminescent substrate to the membrane. This reaction produces light that can be detected using an imaging system. Image Capture and Analysis: Utilize an imaging system to capture the chemiluminescent signal. Analyze the intensity of the protein bands using appropriate software. Compare the target protein band intensity with control or reference samples.

### Statistical methods

The results are presented as the mean ± standard error of the mean. Fisher’s test was used for categorical variables. For continuous variables, an independent-samples *t*-test was employed; when equal variances were not assumed, the Brown–Forsythe test was performed. All statistical analyses were conducted using SPSS software, version 24.0 (IBM Corp., Armonk, NY, USA). A *p*-value < 0.05 was considered statistically significant.

### Data availability

The data used to support the findings of this study are available from the corresponding author upon request.

## RESULTS

### Differential gene analysis

**Figure 1:**
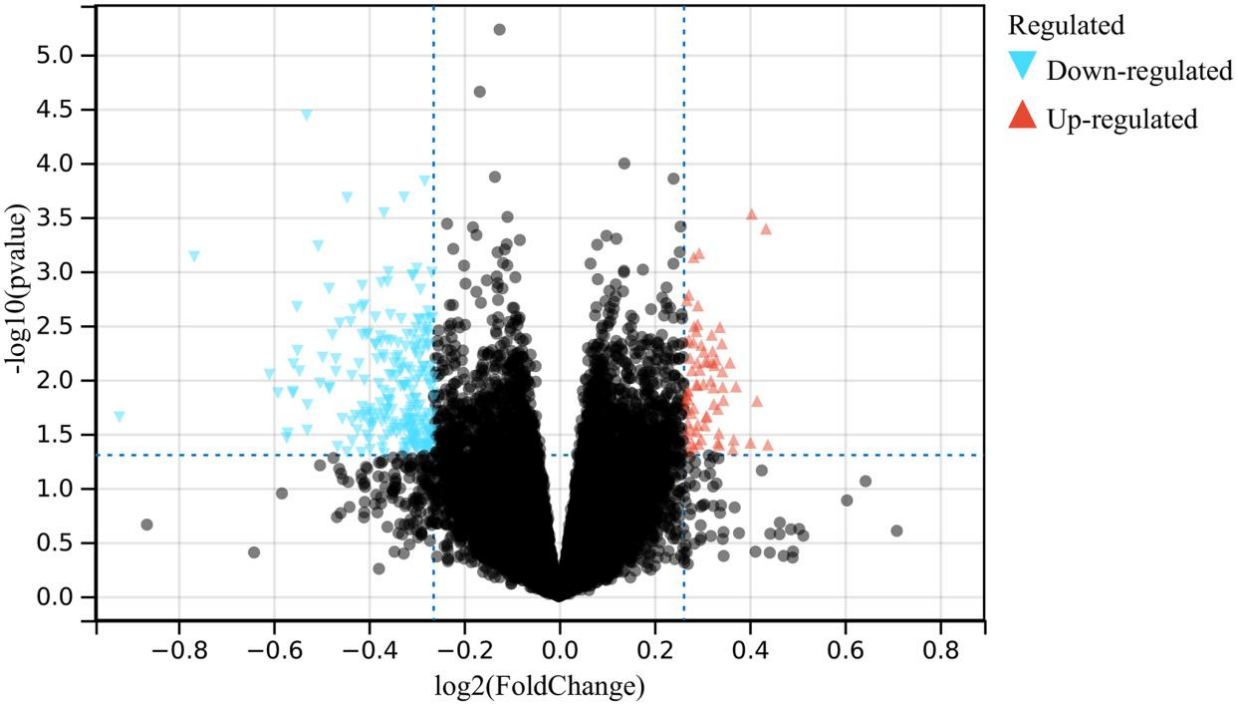
The volcano plot presenting the DEGs of hypertension.

In this study, a total of 500 differentially expressed genes (DEGs) were identified according to the batch removal merge matrix of GSE75672 (Figure 1).

### Functional enrichment analysis

#### Differentially expressed genes

**Figure 2:**
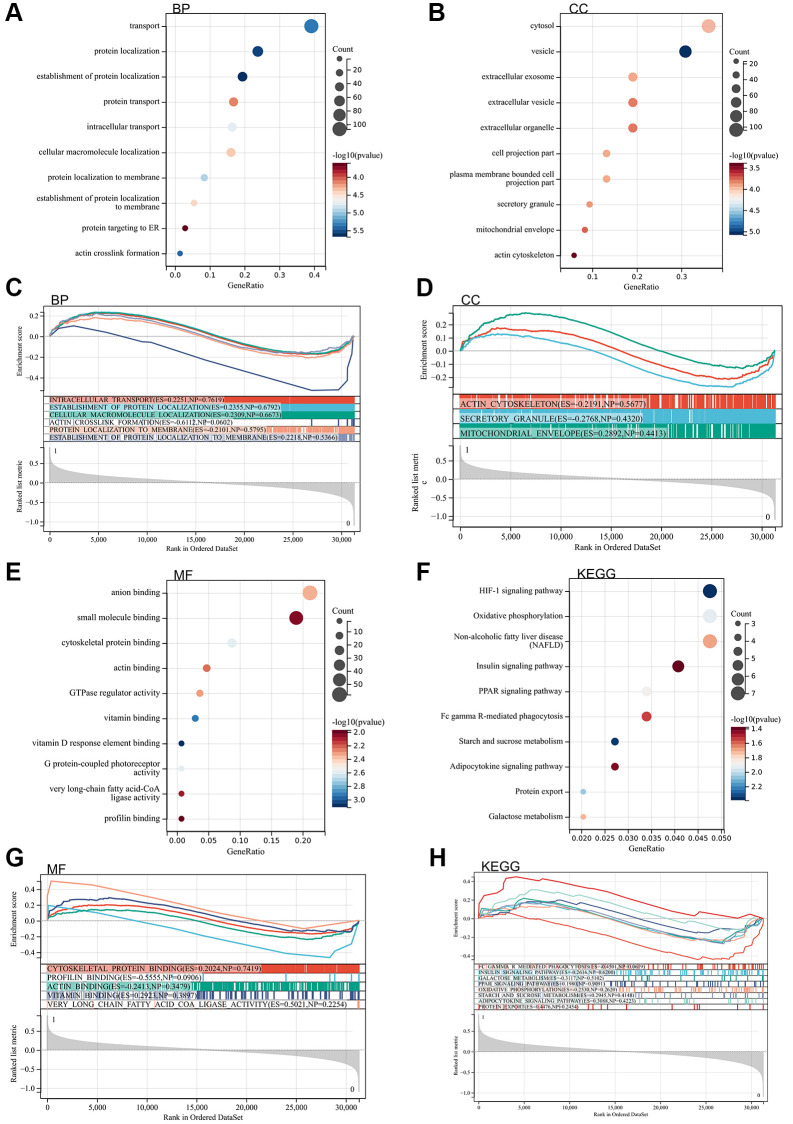
**The enrichment analysis of the DEGs.** (**A**) BP analysis by the DAVID. (**B**) CC analysis by the DAVID. (**C**) BP analysis by the GSEA. (**D**) CC analysis by the GSEA. (**E**) MF analysis by the DAVID. (**F**) KEGG analysis by the DAVID. (**G**) MF analysis by the GSEA. (**H**) KEGG analysis by the GSEA.

Subsequently, Gene Ontology Analysis (GO) and Kyoto Encyclopedia of Genes and Genomes analysis (KEGG) analysis are conducted on these differentially expressed genes. According to the Gene Ontology Analysis (GO) analysis, their primary concentrated is in protein localization, ER-targeted protein, HIF-1 signaling pathway, insulin signaling pathway, PPAR signaling pathway, Adipocytokine signaling pathway, and motor protein binding (Figure 2A–2H).

#### Gene set enrichment

Furthermore, gene set enrichment (GSEA) enrichment analysis was performed on the entire genome to identify potential enrichment items in non-differentially expressed genes. The results, as depicted in the figure, revealed enrichment items similar to those in the Gene Ontology Analysis (GO) and Kyoto Encyclopedia of Genes and Genomes analysis (KEGG) analyses of differentially expressed genes. The concentration is primarily in actin cross-linking formation, insulin signaling pathway, PPAR signaling pathway, Adipocytokine signaling pathway, and protein outlet (Figure 2A–2H).

#### Metascape enrichment analysis

**Figure 3:**
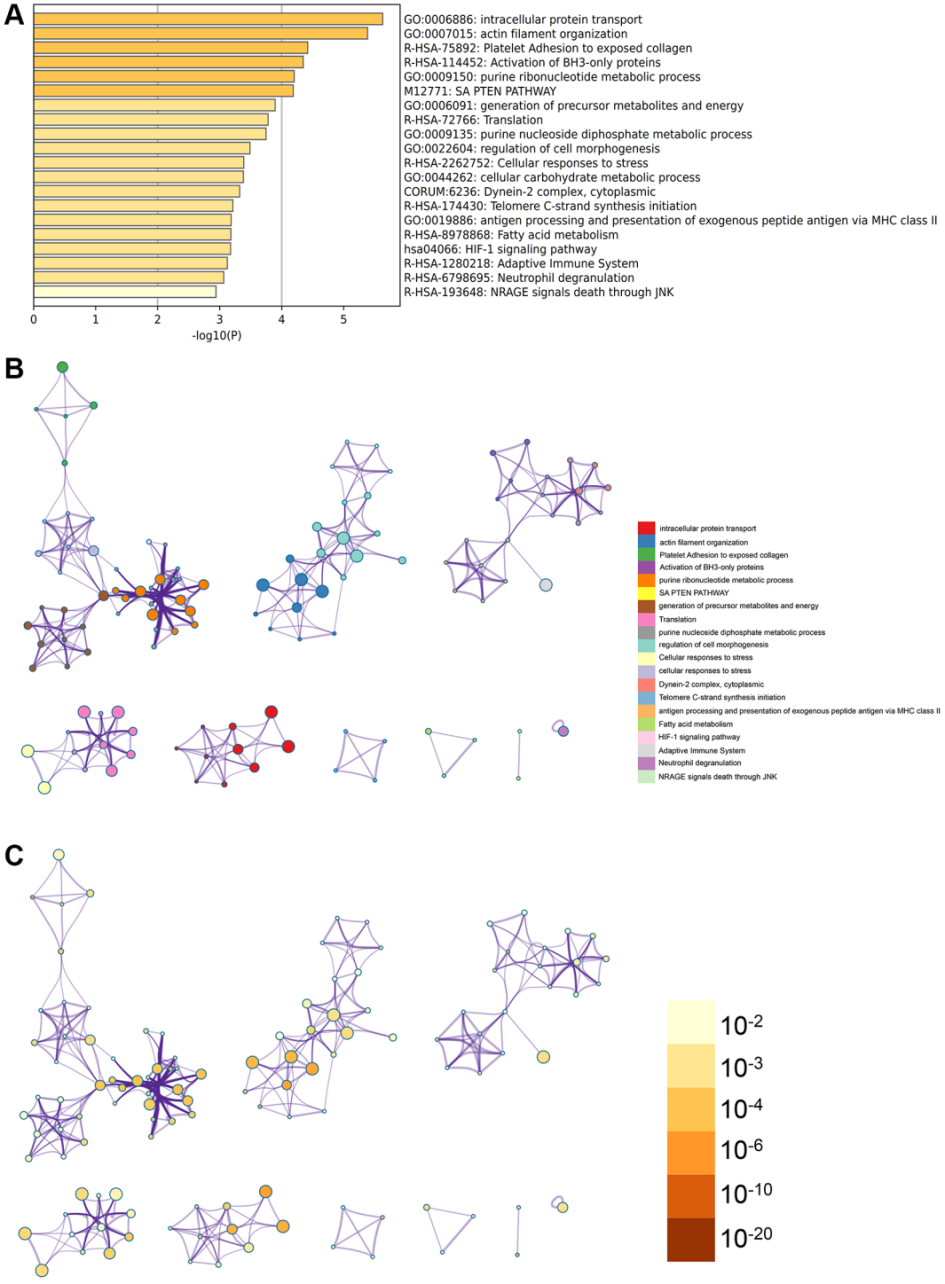
**Metascape analysis for the DEGs.** (**A**) The DEGs were enriched in the cellular carbohydrate metabolic process, intracellular protein transport. (**B**) The interact network of the enrichment terms. (**C**) The enrichment terms were of significance.

**Figure 4:**
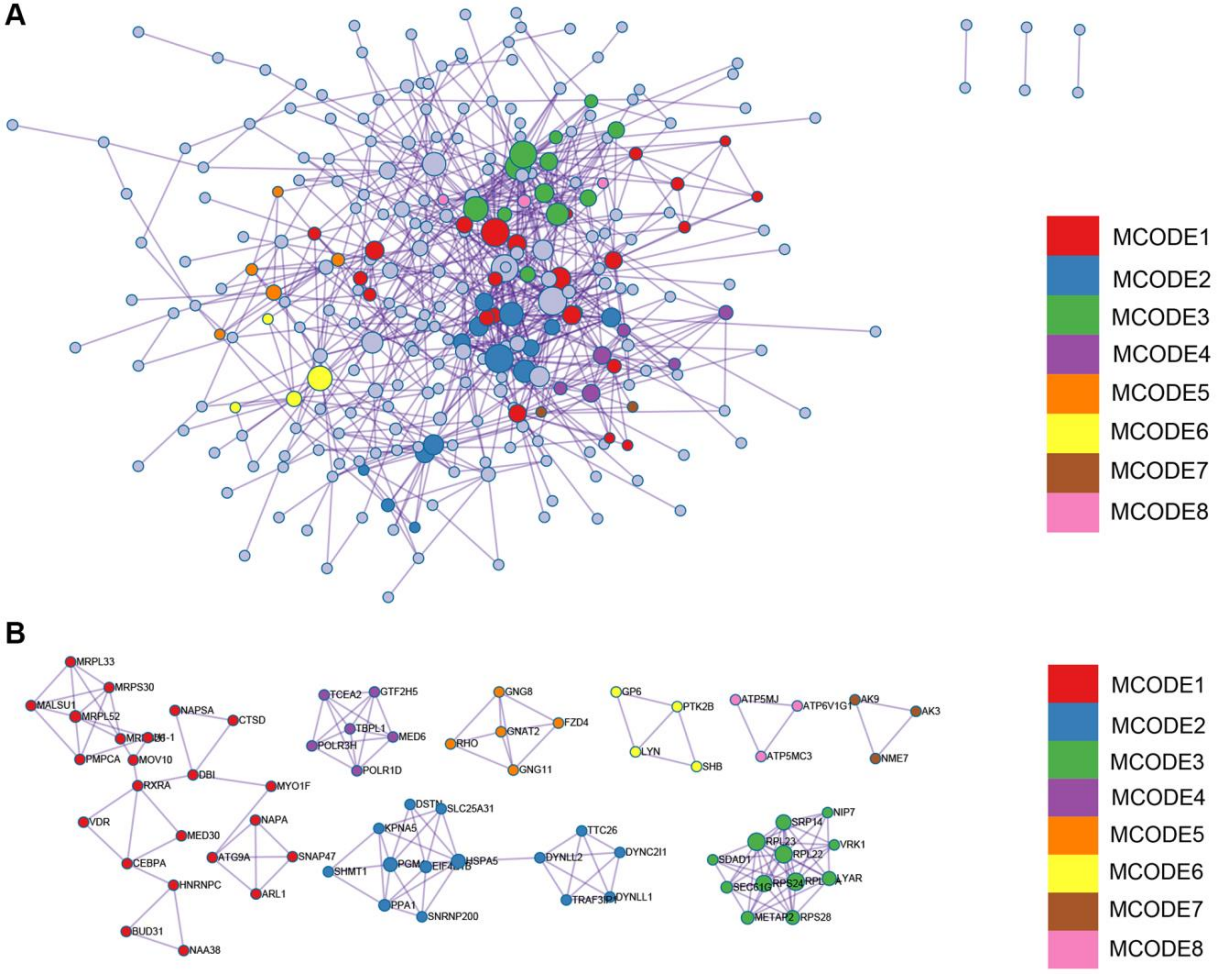
**The significant modules of enrichment terms.** (**A**) protein-protein interact network of the DEGs divided into different modules. (**B**) MCODE networks.

In the Metascape enrichment analysis, the Gene Ontology (GO) results include the SAPTEN pathway, HIF-1 signaling pathway, and activation of BH3 only protein (Figure 3A). The enrichment network is illustrated with term coloring and *P*-value coloring (Figures 3B, 3C and 4).

### Weighted gene coexpression network analysis

**Figure 5:**
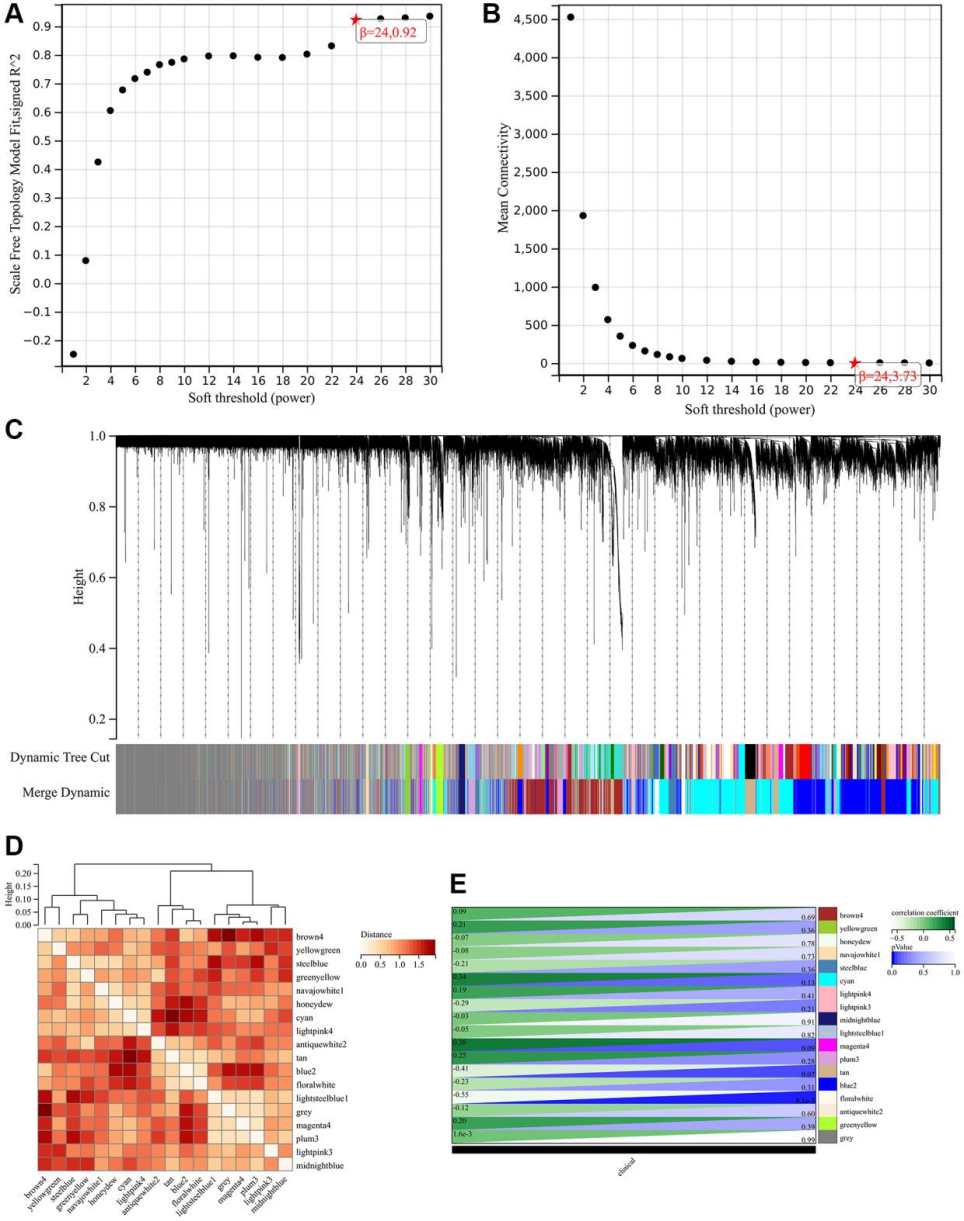
**Weighted gene co-expression network analysis to identify hub genes of the hypertension.** (**A**, **B**) Soft threshold (power) and the β = 24. (**C**) Dynamic tree cut. (**D**) The correlation among the different modules. (**E**) The relationships between hypertension and significant modules.

The selection of soft threshold power is a crucial step in Weighted Gene Coexpression Network Analysis (WGCNA) analysis. To determine the soft threshold power, a network topology analysis was performed. In the Weighted Gene Coexpression Network Analysis (WGCNA) analysis, the soft threshold power is set to 9, representing the lowest power for achieving a scale-free topological fitting index of 0.9 (Figure 5A, 5B). Hierarchical clustering trees were constructed for all genes, resulting in the generation of 18 important modules (Figure 5C). We calculated the module characteristic vector correlation with the expression of genes for MM, according to the cutting standard (MM | | > 0.8), in addition we also merge the distance is less than 0.25 module, eventually won 18 expressing module. The interactions between these modules are then analyzed (Figure 5D). A heat map of the correlation between modules and phenotypes was generated (Figure 5E).

### Construction and analysis of protein-protein interaction network

**Figure 6:**
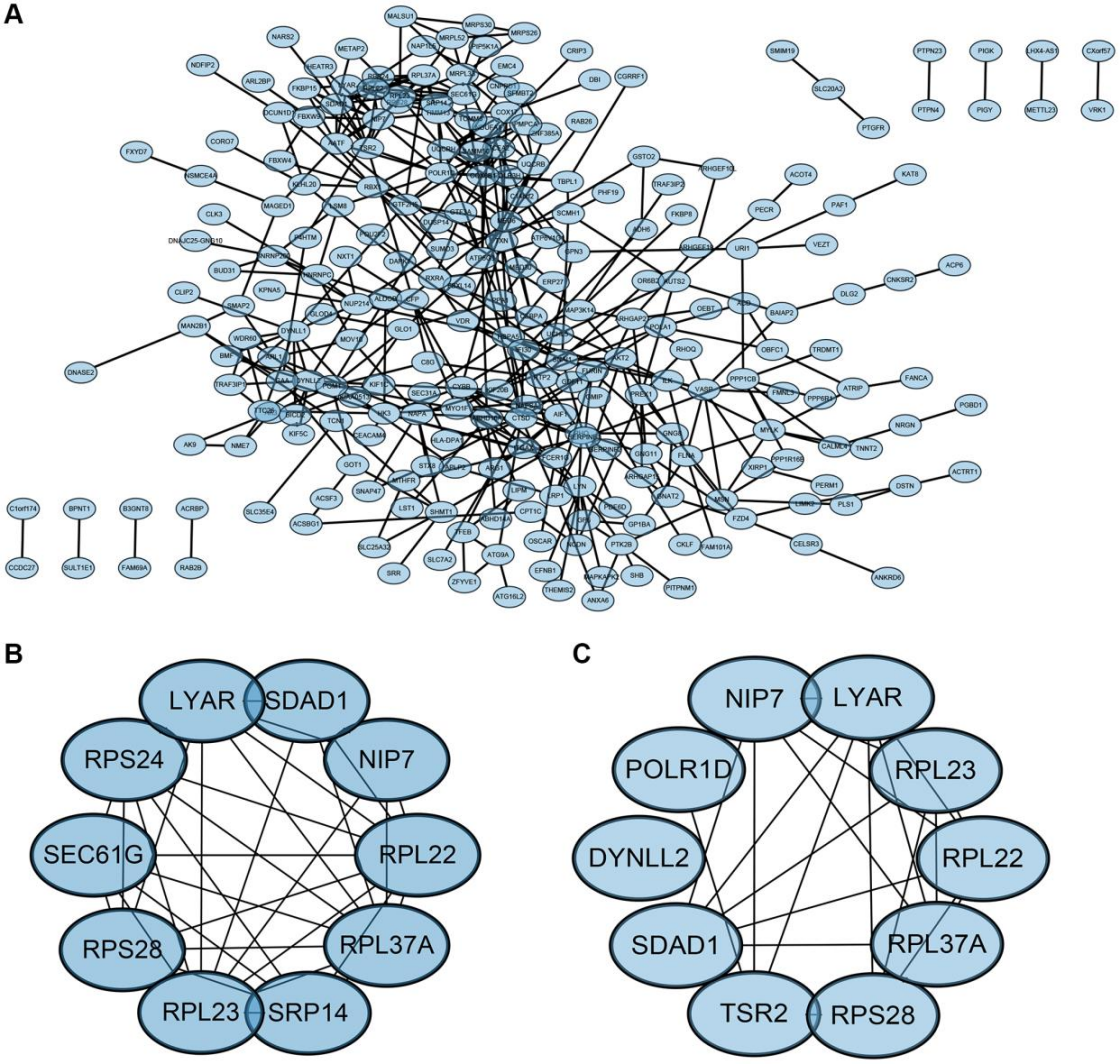
**PPI network and the identification of hub genes.** (**A**) PPI. (**B**, **C**) Two different algorithms to identify central genes.

The Protein-Protein Interaction (PPI) network of Differentially Expressed Genes (DEGs) was constructed using the Search Tool for the Retrieval of Interacting Genes (STRING) online database and analyzed with Cytoscape software (Figure 6A). Two different algorithms were employed to identify central genes (Figure 6B, 6C). Considering the research significance, we have excluded ribosomal genes. Finally, eight core genes (SEC61G, SRP14, Liy AR, NIP7, SDAD1, POLR1D, DYNLL2, TSR2) were obtained.

### Gene expression heat map

**Figure 7:**
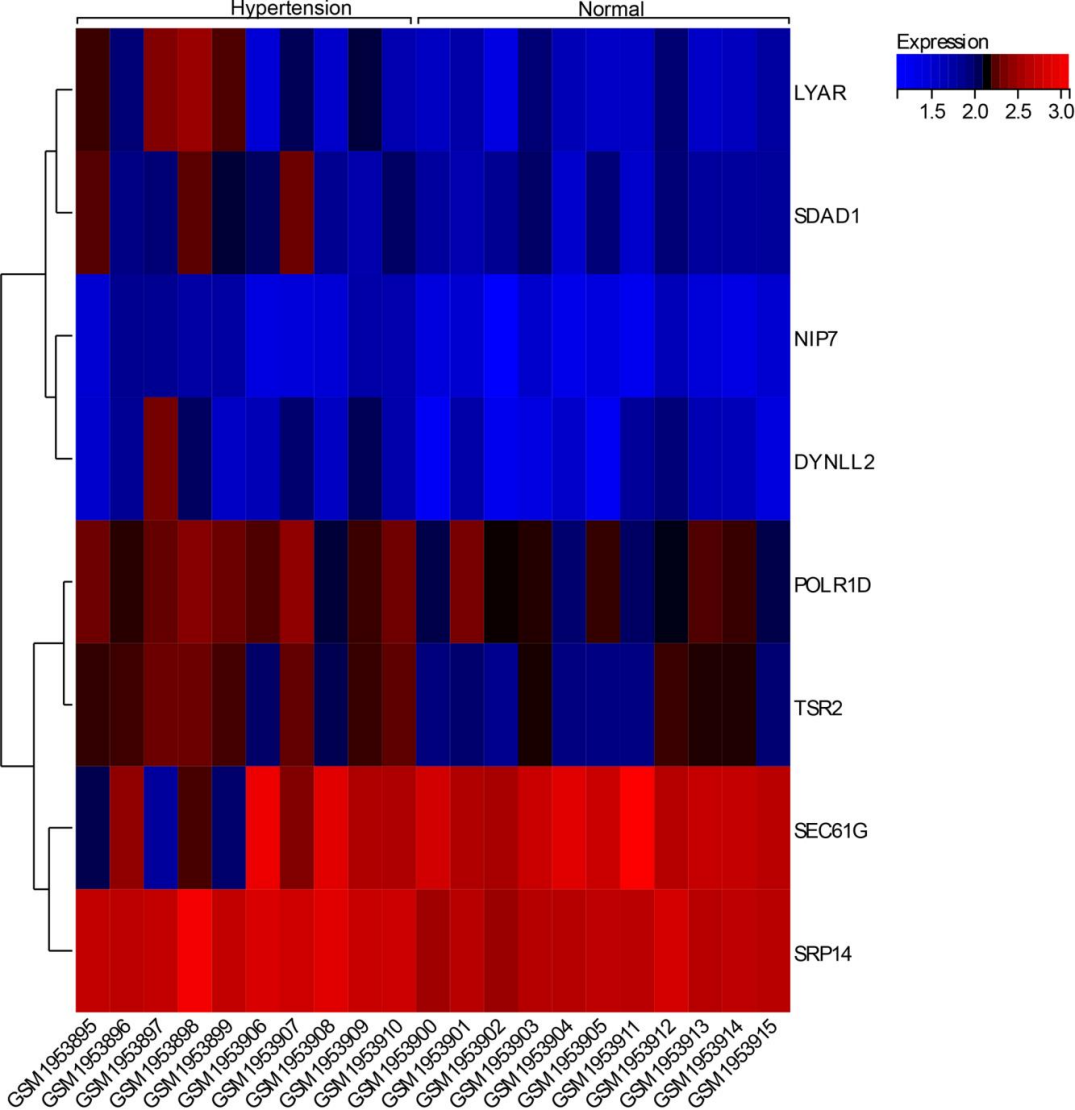
Expression calorigram of hub genes in the samples.

We visualized the expression dendrogram of core genes in the samples (Figure 7) and observed that four core genes (LYAR, SDAD1, POLR1D, TSR2) exhibited high expressed levels in the hypertension tissue samples, whereas they were low levels in the normal tissue samples. This suggests a potential regulatory effect on hypertension associated with these genes.

### Comparative toxicogenomics database analysis

**Figure 8:**
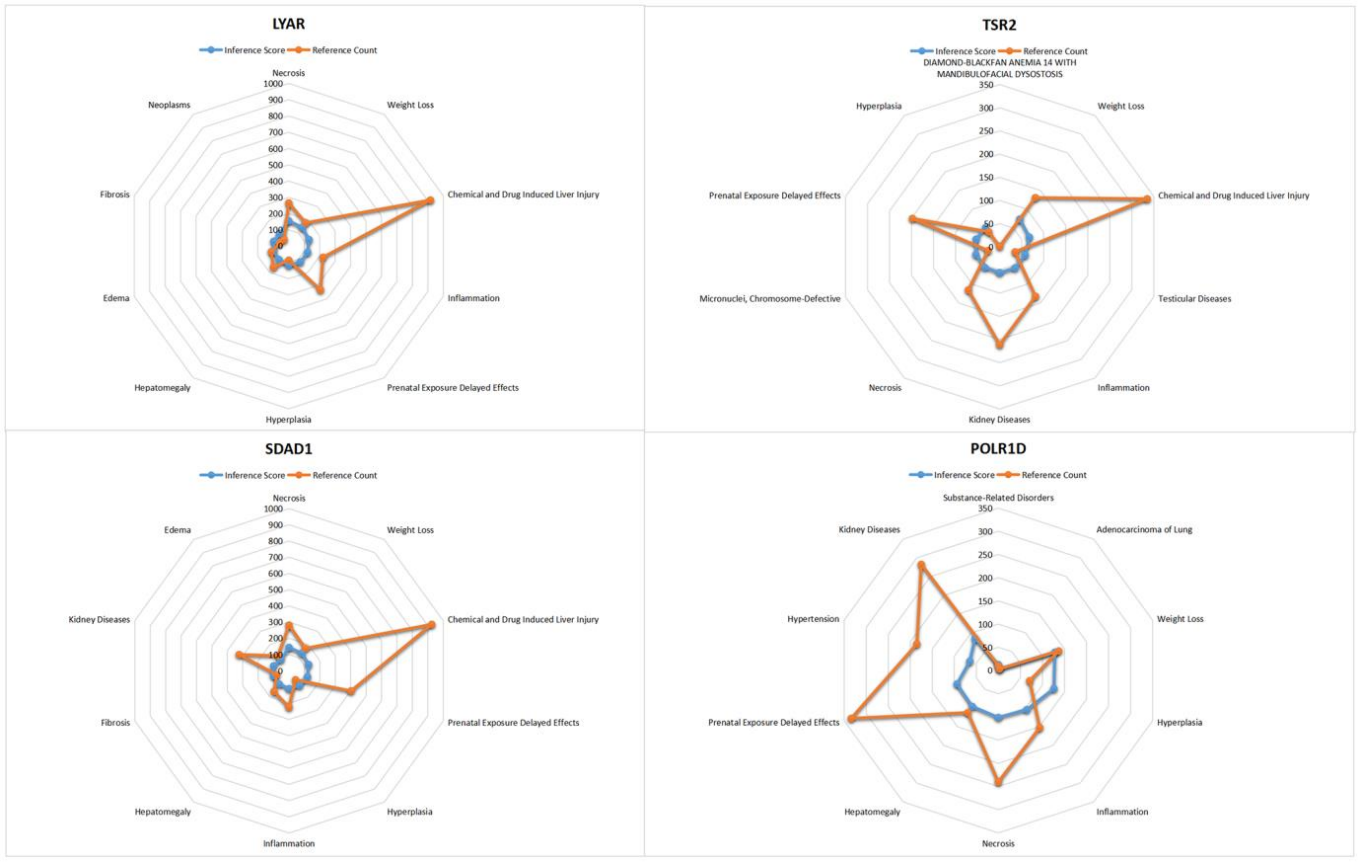
CTD analysis for the LYAR, TSR2, SDAD1, POLR1D.

In this study, we entered the list of hub genes into the Comparative Toxicogenomics Database (CTD) website to explore diseases associated with core genes, enhancing our understanding of the association between genes and diseases. Four genes (LYAR, SDAD1, POLR1D, TSR2) were found to be associated with hypertension, edema, inflammation, and Diamond-Blackfan anemia (see Figure 8).

### Prediction and functional annotation of miRNA associated with hub genes

In this study, the hub gene list was input into TargetScan to search for related miRNA, enhancing our understanding of gene expression regulation (Table 1). We identified that the related miRNA of SDAD1 gene were hsa-miR-30d-5p, hsa-miR-30e-5p and hsa-miR-30b-5p. The miRNA associated with POLR1D gene was hsa-miR-216b-5p. TSR2 gene related miRNA were hsa-miR-135b-5p and hsa-miR-135a-5p.

**Table 1:** A summary of miRNAs that regulate hub genes.

	**Gene**	**MIRNA**		
**1**	**SDAD1**	hsa-miR-30d-5p	hsa-miR-30e-5p	hsa-miR-30b-5p
**2**	**POLR1D**	hsa-miR-216b-5p		
**3**	**TSR2**	hsa-miR-135b-5p	hsa-miR-135a-5p	
**4**	**LYAR**	none		

### The verification of expression of TSR2

Compared with the normal peripheral blood mononuclear cells (PBMC), the expression of TSR2 was up-regulated in the hypertension peripheral blood mononuclear cells (PBMC) (*P* < 0.01). Through the western blotting assay, the expression of TSR2 in the hypertension peripheral blood mononuclear cells (PBMC) + TSR2_ KD was lower than the hypertension peripheral blood mononuclear cells (PBMC). And the expression of TSR2 in the hypertension peripheral blood mononuclear cells (PBMC) + TSR2_ OV was higher than the hypertension peripheral blood mononuclear cells (PBMC). The knock down assay and over expression assay were successful (*P* < 0.05).

### TSR2 might active the PPAR signaling pathway

When TSR2 was higher in the hypertension peripheral blood mononuclear cells (PBMC)than normal peripheral blood mononuclear cells (PBMC), the critical proteins in the PPAR signaling pathway (FABP, PPAR, PLTP, ME1, SCD1, CYP27, FABP1, OLR1, CPT-1, PGAR, CAP, ADIPO, MMP1, UCP1, ILK, PDK1 UBC AQP7) were also higher in the hypertension peripheral blood mononuclear cells (PBMC). When TSR2 was knocked down in the hypertension peripheral blood mononuclear cells (PBMC) model, the critical proteins in the PPAR signaling pathway (FABP, PPAR, PLTP, ME1, SCD1, CYP27, FABP1, OLR1, CPT-1, PGAR, CAP, ADIPO, MMP1, UCP1, ILK, PDK1 UBC AQP7) were downregulated. This also occurred in the hypertension peripheral blood mononuclear cells (PBMC) + TSR2_ OV model. When TSR2 was over expressed in the hypertension peripheral blood mononuclear cells (PBMC), the critical proteins in the PPAR signaling pathway (FABP, PPAR, PLTP, ME1, SCD1, CYP27, FABP1, OLR1, CPT-1, PGAR, CAP, ADIPO, MMP1, UCP1, ILK, PDK1 UBC AQP7) were also higher in the hypertension peripheral blood mononuclear cells (PBMC) + TSR2_ OV.

**Figure 9:**
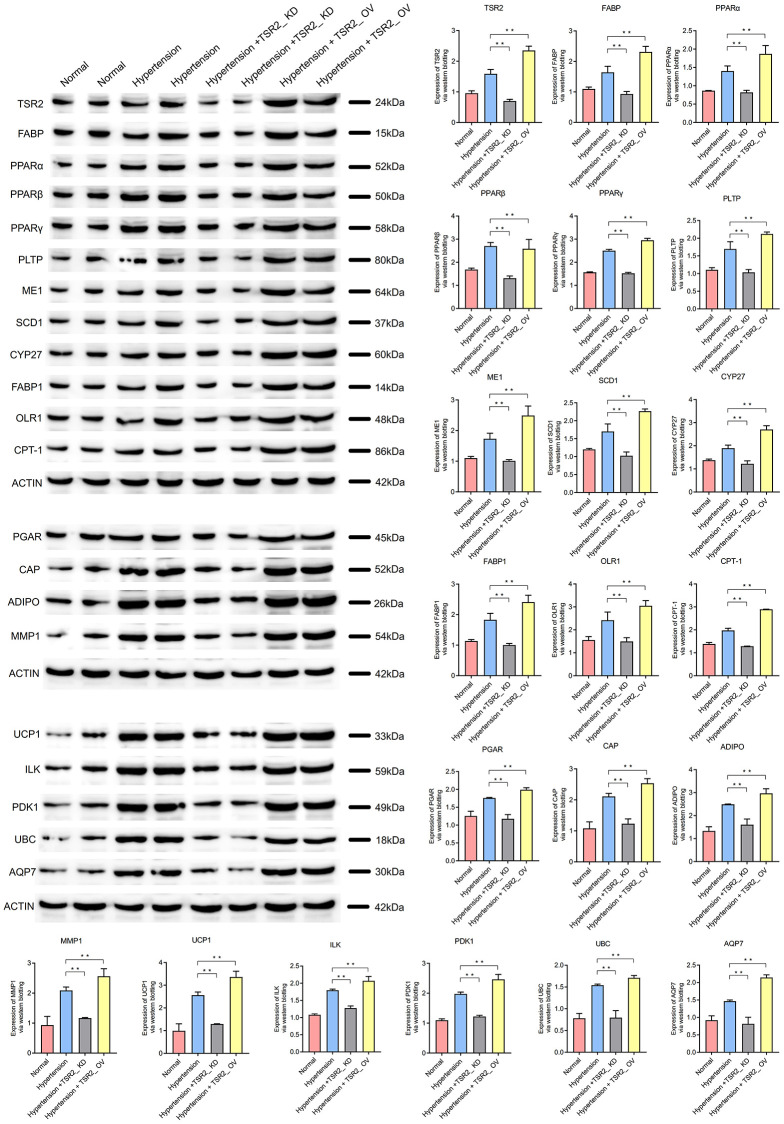
TSR2 might active the PPAR signaling pathway to affect the hypertension.

These results showed that TSR2 might activate the PPAR signaling pathway and affect hypertension. (Figure 9).

## DISCUSSION

The genetic factor in the pathogenesis of essential hypertension, namely family relationship, has been confirmed through many years of medical practice [16, 17]. The familial tendency of hypertension was established early in life, and the incidence of hypertension in the children of both parents was 46%. If one parent suffers from hypertension, the incidence in their children is 28%, versus only 3% in children born to parents with normal blood pressure. In studies on susceptibility genes for essential hypertension, the number of identified susceptibility genes has increased to dozens [18, 19]. In-depth exploration of the molecular mechanism of hypertension is crucial for targeted drug research [20]. The primary outcome of this study was that TSR2 was highly expressed in hypertension and promoted the activation of PPAR signaling pathway. When TSR2 is overexpressed, it can activate the PPAR signaling pathway and promote the occurrence and development of hypertension.

TSR2 plays a crucial role in the activation of TGF-β1. TSR2 is a basic sequence of TSP-1. TSP1 is a homotrimer matrix glycoprotein with a molecular weight of 450 kDa, composed of three identical peptide chains. Each peptide chain consists of n-terminal and C-terminal globular domains, as well as type1, type2, and type3 homologous regions of procollagen. Type1 repeats include TSR1, TSR2 and TSR3 [21]. TSP-1 specifically binds to L-TGF-β1 through the KRFK amino acid domain between TSR1 and TSR2, forming the TSP-1/L-TGF-β1 protein complex. Subsequently, this complex specifically binds to the CD36 receptor of TSP-1 on the cell membrane through the CSVTCG amino acid peptide domain in the TSR2 and TSR3 motifs. This interaction induces a spatial conformational change in L-TGF-β1, leading to the separation of TGF-β1 and LAP and the production of activated TGF-β1 [22]. All TSR1, TSR2 and TSR3 contain WSXW sequences, enabling them to interact with the LAP in L-TGF-β1. This interaction promotes the specific binding of the KRFK amino acid domain between TSR1 and TSR2 with L-TGF-β1, ultimately generating activated TGF-β1 [23].

The Comparative Toxicogenomics Database (CTD) analysis results in this study suggest that the core genes are primarily associated with hypertension, edema, inflammation, and Diamond-Blackfan anemia. It was observed that overexpression of TSR2 may disrupt the NF-κB signaling pathway by reducing nuclear NF-κB p65 and increasing cytoplasmic NF-κB p65. Additionally, TSR2 overexpression significantly inhibits the phosphorylation of IκBα and IKKα/β, contributing to the development of inflammatory diseases [24]. Findings from Yang’s research revealed that Tsr2 releases Rps26 under conditions of high Na+ or pH *in vitro*. Tsr2 stores free Rps26 and promotes the reintegration of proteins, thereby facilitating the repair of subunits after the Na+ stress subsides. Residues in Rps26 show a strong correlation with Diamond-Blackfan anemia in response to Na+ stress [25]. Furthermore, the association with hypertension involves TGF-β1, considered a key fibrogenic factor and one of the most potent inducers of extracellular matrix deposition discovered to date [26–28]. Previous research has demonstrated that the TGF-β1/Smad3 pathway regulates macrophage polarization and improves the mechanism of radiation-induced lung fibrosis [29]. In Calvier’s [30] study, it was found that PPARγ connects the BMP2 and TGFβ1 pathways in vascular smooth muscle cells, regulating cell proliferation and glucose metabolism. Therefore, we speculate that TSR2 may induce vascular fibrosis and consequently contribute to the development of hypertension by modulating TGF-β1.

The results of the functional enrichment analysis in this study demonstrate significant enrichment in terms of protein localization, ER-targeted proteins, the HIF-1 signaling pathway, the insulin signaling pathway, the PPAR signaling pathway, the Adipocytokine signaling pathway, and motor protein binding. These demonstrations are all related to the function and regulation of proteins. While TSR2 gene expression is influenced by various pathways in its effects, the role of PPAR seems to be predominant.

Activation of PPAR leads to a substantial increase in intracellular lipid droplets and an enlargement of their volume, accompanied by an approximately 20% rise in lipid content. The elevation in intracellular Reactive Oxygen Species (ROS) levels induced by oxidative stress, a byproduct of peroxisomal fatty acid oxidation, is typically associated with cellular aging or lipid peroxidation networks [31]. Song’s study [32] found that the PPAR-α/AMPK pathway inhibits oxidative stress and modulates energy metabolism, thereby alleviating diabetes-induced cardiomyopathy.

PPAR induces peroxisome proliferation, ultimately resulting in a state of intracellular peroxidation. Traditionally recognized as scavengers of Reactive Oxygen Species (ROS), peroxisomes remove hydrogen peroxide produced during peroxisomal Fatty Acid Oxidation (FAO) through their internal Glutathione peroxidase (GPX), Catalase (CAT), and Superoxide Dismutase (SOD) 1/2 enzymes. They play a crucial role in clearing excess Reactive Oxygen Species (ROS) within cells and maintaining intracellular redox homeostasis [33, 34]. Khundmiri’s research [35] demonstrated that PPAR-α may attenuate angiotensin II-induced blood pressure elevation by reducing the expression and activity of NKA through mouse PPAR gene knockout and overexpression experiments. A recent study also elucidated that Chrysophyllum albidum fruit ethanol extract improves hyperglycemia and hypertension in streptozotocin-induced diabetic rats by regulating oxidative stress, NF-κB, and PPAR-γ [36].

Activating peroxisome proliferators-activated receptors (PPARs) activates the biosynthesis process of peroxisome. In this process, the active oxidation of fatty acids in peroxisome leads to an increase in H_2_O_2_, a metabolic by-product [37]. However, the varied responses of downstream molecules to this signal result in a decreased affinity of PEXS and Catalase (CAT) in newborn peroxisome [38]. Some newborn peroxisome lose the ability to clear H_2_O_2_ due to the absence of Catalase (CAT). Consequently, the overactivity of Reactive Oxygen Species (ROS) generation and the weakening of Reactive Oxygen Species (ROS) scavenging ability transform peroxisome change from Reactive Oxygen Species (ROS) scavengers to producers in functional state [39]. Therefore, it is speculated that PPAR signaling pathway may play a crucial role in the growth and development of hypertension. As a protein, TSR2 participates in the intricate processes of protein synthesis and translation within cells. These processes intricately intertwine with gene expression within the PPAR pathway. PPAR nuclear receptors can regulate lipid and glucose metabolism by activating or inhibiting the transcription of specific genes [40, 41]. Furthermore, the actions of TSR2 may exert influence on immune and inflammatory processes, and it’s noteworthy that the PPAR pathway is intricately associated with immune and inflammatory responses. This suggests a potential interconnection between TSR2 and the role of the PPAR pathway in these vital biological processes.

Therefore, TSR2 may impact lipid metabolism by influencing the expression or activity of relevant genes within the PPAR pathway. Dysregulation of lipid metabolism can lead to the development of hypertension. Moreover, TSR2 may modulate inflammatory and immune responses by influencing gene expression or activity in the PPAR pathway, thereby influencing hypertension. Additionally, the actions of TSR2 may affect oxidative stress by influencing the expression of genes associated with oxidative stress within the PPAR pathway. Oxidative stress is a phenomenon related to the disturbance of the cellular redox balance and may be linked to the development of hypertension. Despite the rigorous bioinformatics analysis, there are still some shortcomings in this paper. No clinical specimen test was performed in this study to further verify its function. Therefore, in the future research, we should conduct in-depth exploration in this aspect.

## CONCLUSION

In summary, TSR2 is overexpressed in individuals with hypertension and may exert an influence on hypertension through the PPAR signaling pathway, affecting lipid metabolism, immune and inflammatory responses, and oxidative stress. TSR2 could potentially serve as a molecular target for early diagnosis and precision treatment of hypertension, providing a foundation for mechanistic research in hypertension.
